# Tyrphostin AG556 increases the activity of large conductance Ca^2+^‐activated K^+^ channels by inhibiting epidermal growth factor receptor tyrosine kinase

**DOI:** 10.1111/jcmm.13103

**Published:** 2017-03-14

**Authors:** Yan Wang, Hai‐Ying Sun, Ying‐Guang Liu, Zheng Song, Gang She, Guo‐Sheng Xiao, Yan Wang, Gui‐Rong Li, Xiu‐Ling Deng

**Affiliations:** ^1^ Department of Physiology and Pathophysiology School of Basic Medical Sciences Xi'an Jiaotong University Health Science Center Xi'an Shaanxi China; ^2^ Department of Medicine Li Ka Shing Faculty of Medicine The University of Hong Kong Hong Kong China; ^3^ Institute of Basic Medical Sciences Xi'an Medical University Xi'an Shaanxi China; ^4^ Xiamen Cardiovascular Hospital Xiamen University Xiamen Fujian China; ^5^ Cardiovascular Research Centre School of Basic Medical Sciences Xi'an Jiaotong University Health Science Center Xi'an Shaanxi China

**Keywords:** epidermal growth factor receptor tyrosine kinase, large conductance Ca^2+^‐activated K^+^ channels, protein tyrosine phosphatase, tyrphostin AG556

## Abstract

The present study was designed to investigate whether large conductance Ca^2+^‐activated K^+^ (BK) channels were regulated by epidermal growth factor (EGF) receptor (EGFR) tyrosine kinase. BK current and channel tyrosine phosphorylation level were measured in BK‐HEK 293 cells expressing both functional α‐subunits and the auxiliary β1‐subunits using electrophysiology, immunoprecipitation and Western blotting approaches, respectively, and the function of rat cerebral basilar arteries was determined with a wire myography system. We found that BK current in BK‐HEK 293 cells was increased by the broad spectrum protein tyrosine kinase (PTK) inhibitor genistein and the selective EGFR tyrosine kinase inhibitor AG556, one of the known tyrphostin. The effect of genistein or AG556 was antagonized by the protein tyrosine phosphatase (PTP) inhibitor orthovanadate. On the other hand, orthovanadate or EGF decreased BK current, and the effect was counteracted by AG556. The tyrosine phosphorylation level of BK channels (α‐ and β1‐subunits) was increased by EGF and orthovanadate, while decreased by genistein and AG556, and the reduced tyrosine phosphorylation of BK channels by genistein or AG556 was reversed by orthovanadate. Interestingly, AG556 induced a remarkable enhancement of BK current in rat cerebral artery smooth muscle cells and relaxation of pre‐contracted rat cerebral basilar arteries with denuded endothelium, and these effects were antagonized by the BK channel blocker paxilline or orthovanadate. These results demonstrate that tyrosine phosphorylation of BK channels by EGFR kinase decreases the channel activity, and inhibition of EGFR kinase by AG556 enhances the channel activity and dilates rat cerebral basilar arteries.

## Introduction

Large‐conductance, voltage‐ and Ca^2+^‐activated K^+^ (BK, also called BK_Ca_ or Maxi K) channels are distributed ubiquitously in mammalian tissues. The basic functional unit of BK channels is a tetramer of pore‐forming α‐subunits (KCa1.1 or Slo1) encoded by *KCNMA1* 1. BK channel α‐subunits interact with auxiliary β‐subunits (β1‐β4) in a tissue type‐dependent manner 2, 3. In vascular smooth muscle, β1 encoded by *KCNMB1* is the predominant subunit associated with α‐subunit and confers BK channels with high sensitivity to Ca^2+^, which makes the channel an efficient tuner of smooth muscle function 4, 5, 6. Besides the membrane potential and intracellular free Ca^2+^, the activity of BK channels is also regulated by intracellular signals including protein phosphorylation, *e.g*. serine and threonine phosphorylation mediated by protein kinase A, protein kinase C, protein kinase G 7, 8, and also protein tyrosine kinases (PTKs), including receptor PTKs such as epidermal growth factor receptor (EGFR) tyrosine kinase and non‐receptor PTKs (*e.g*. Src‐family kinases, Janus activated kinase 2 and 3) 9, 10, 11, 12, 13, 14.

However, the reports on the regulation of BK channels by PTKs are controversial. The non‐receptor PTK c‐Src is found to inhibit BK channel activity in vascular smooth muscle cells (VSMCs) 15, but to enhance BK channel activity in a heterologous expression system 11, 16. On the other hand, the broad spectrum PTK inhibitor genistein and the EGFR tyrosine kinase inhibitor tyrphostin 51 increase BK current in the cultured cells from bovine trabecular meshwork, a smooth muscle‐like tissue involved in the regulation of aqueous humor outflow 9, while genistein inhibits the BK activation in VSMCs from rats with hemorrhagic shock 10. Therefore, the regulation of BK channels by PTKs still remains to be clarified.

Tyrphostins, known as analogs (AG) compounds, are a series of synthetic low molecular weight antiproliferative compounds that act as PTK inhibitors by binding to the substrate binding site. Many of the tyrphostins have selective and distinct inhibitory activities in various tyrosine kinase assay systems. AG556, one of the most widely used tyrphostin in different studies 17, 18, 19, is a selective EGFR tyrosine kinase inhibitor 20. AG556 exhibits pronounced beneficial effects in an induced myocardial infarction model 21 and in experimental autoimmune myocarditis 22 in rats, as well as in a mouse model of arterial injury 23, suggesting that AG556 possibly is effective in attenuating pathophysiological conditions. In the present study, we investigated the effects of AG556 and genistein on BK channels, and explored the involvement of EGFR tyrosine kinases. We found that the inhibition of EGFR tyrosine kinase by AG556 or genistein enhanced BK channel activity in BK‐HEK 293 cells stably expressing both α‐ and β1‐subunits of human BK channels. In addition, AG556 increased BK channel current in isolated rat cerebral artery smooth muscle cells (CASMCs) and dilated rat cerebral basilar arteries.

## Materials and methods

### Cell culture

Human BK channel α and the β1 pcDNA3.1 plasmids provided by Dr. Christopher J. Lingle (Washington University, St. Louis, MO, USA) were transfected separately into HEK 293 cells (ATCC; Manassas, VA, USA) in a 35‐mm culture dish with Lipofectamine 2000 (Invitrogen, Hong Kong, China) to establish BK‐HEK 293 cells stably expressing both α‐ and β1‐subunits. The cells were cultured in Dulbecco's modified Eagle's medium (Invitrogen) supplemented with 10% fetal bovine serum (Invitrogen) and 400 mg/ml G418 (Invitrogen). Cells used for electrophysiology were seeded on glass cover slips.

### Animal preparation

All experimental protocols were approved by the Institutional Animal Care and Use Committee of Xi'an Jiaotong University and conformed to the Guide for the Care and Use of Laboratory Animals published by the National Institutes of Health. The experiment was conducted on a cohort of adult male Sprague‐Dawley rats (200 ± 18 g) obtained from the Laboratory Animal Centre of Xi'an Jiaotong University. All animals were housed under conditions of 22 ± 2°C, 55 ± 5% humidity, and a 12‐hrs light/dark cycle and allowed *ad libitum* access to water and a common laboratory rodent chow.

### Isolation of cerebral basilar arteries and vascular tension measurement

After the rats were anesthetized with sodium pentobarbital (50 mg/kg i.p.), the cerebral basilar arteries were carefully isolated from the brain under a dissecting microscope, and immediately placed in ice‐cold Krebs–Henseleit solution (KHS, pH 7.4) gassed with a mixture of 95% O_2_ and 5% CO_2_. The KHS composition contained (mM): NaCl 115, NaHCO_3_ 25, KCl 4.6, NaH_2_PO_4_ 1.2, MgCl_2_ 1.2, CaCl_2_ 2.5, and glucose 10. Each basilar artery was separated from the surrounding connective tissues, and cut into 3‐mm long rings without endothelium which was denuded by gently rubbing the intimal surface of the vessel with a human hair 24, 25. The arterial rings were threaded onto two stainless steel wires (40 μm in diameter) and mounted in 5‐ml chambers of a multi‐wire myograph system (model 610M; Danish Myo Technology, Aarhus, Denmark) containing KHS continuously aerated with 95% O_2_ and 5% CO_2_ at 37°C for isometric force measurements. Tension signals were relayed to a PowerLab recording unit and saved to a Chart 7 for Windows software (AD Instruments Ltd, Aarhus, Denmark). The vessels were then allowed to equilibrate for at least 60 min. with the bath solution changed every 15 min. After the equilibration, reactivity of the rings was checked thrice by administration of 60‐mM KCl (achieved by substitution of NaCl in KHS with an equimolar concentration of KCl). To assess the success of endothelium removal, cerebral basilar arteries were precontracted with 1 μM 5‐Hydroxytryptamine (5‐HT), and 10 μM acetylcholine (ACh) as described previously 26 was used to relax the artery rings. ACh‐induced relaxation was <20% of the precontracted tone in all cases, indicating that the endothelium was successfully removed. After washout, the vessels were incubated for 15 min. without or with 1 μM paxilline or 1 mM orthovanadate prior to inducing contraction with 1 μM 5‐HT, then relaxed with 0.01–10 μM AG556. Percentage values of relaxation by AG556 were measured as a percentage of precontraction with application of 5‐HT.

### Isolation of cerebral artery smooth muscle cells

Single CASMCs were isolated enzymatically as described previously 24, 25. In brief, the cerebral arteries were separated from connective and fat tissues, then cut into 1‐mm strips in ice‐cold physiological salt solution (PSS, pH 7.4) containing (mM): NaCl 137, KCl 5.6, MgCl_2_ 1, Na_2_HPO_4_ 0.42, NaH_2_PO_4_ 0.44, NaHCO_3_ 4.2, and 4‐(2‐hydroxyethyl)‐1‐piperazineethanesulfonic acid (HEPES) 10, bubbled with 95% O_2_ and 5% CO_2_. The strips were digested in PSS with 5 mg/ml papain, 2 mg/ml dithioerythritol, and 1 mg/ml bovine serum albumin (BSA) at 37°C for 18 min. After removal of the enzyme solution, the strips were gently triturated with a pipette in enzyme‐free PSS containing BSA to release single CASMC. The suspension was stored at 4°C for use within 6 hrs.

### Electrophysiology

Cells on a coverslip were transferred to a cell chamber (0.5 ml) mounted on the stage of an inverted microscope (Diaphot; Nikon, Tokyo, Japan) and superfused at ~2 ml/min with Tyrode's solution. Whole cell currents were recorded as described previously 27. Borosilicate glass electrodes [1.2‐mm OD (outside diameter)] were pulled with a Brown‐Flaming puller (model P‐97; Sutter Instruments Co, Novato, CA, USA) and had a tip resistance of 2–3 MΩ when filled with the pipette solution. A 3‐M KCl agar bridge was used as the reference electrode. The tip potential was zeroed before the patch pipette contacted the cell. After a giga‐Ohm seal was obtained, the cell membrane was ruptured by applying a gentle negative pressure to establish the whole‐cell configuration. Series resistance was 3–6 MΩ and was compensated for by 50–80% to minimize voltage errors. Membrane currents were measured using an EPC‐10 amplifier and Pulse software (Heka Elektronik GmbH, Lambrecht, Germany). Command pulses were generated by a 12‐bit digital‐to‐analogue converter controlled by Pulse software. Current signals were low‐pass filtered at 5 kHz and stored in the hard disk of an IBM compatible computer. All experiments were conducted at room temperature (22–23°C).

### Immunoprecipitation and Western blotting

The immunoprecipitation and Western blotting experiments were performed following the procedure described previously 17, 28. BK‐HEK 293 cells (~80% confluence) were treated respectively with different compounds, *e.g*. genistein, AG556, orthovanadate and EGF, for 30 min. at room temperature, and centrifuged at 4°C. The cell pellet was then lysed with a lysis buffer containing 25 mM Tris/HCl, 150 mM NaCl, 1.0 mM NaF, 1.0 mM EDTA, 1.0 mM orthovanadate, 1.0 mM PMSF and 1% sodium deoxycholate, 0.1% SDS, 1% Triton X‐100, 1 μg/ml leupeptin and 1 μg/ml aprotinin. Protein quantification of lysates was made using a protein assay reader (Bio‐Rad Laboratories, NY, USA), and diluted to equal concentrations. Proteins were immunoprecipitated overnight at 4°C using 1 μg of mouse anti‐BK channel α (APC‐021; Alomone Labs, Jerusalem, Israel) antibody or 1 μg of mouse anti‐β1 (APC‐036; Alomone Labs, Jerusalem, Israel) antibody and 20 μl of Protein A/G beads (sc‐2003; Santa Cruz Biotechnology, Inc. CA, USA). The immunoprecipitated proteins bound to pelleted Protein A/G beads were washed thoroughly in PBS, denatured in Laemmli sample buffer, separated by sodium dodecyl sulfate/polyacrylamide gel and electroblotted onto nitrocellulose membranes. The immunoblots were probed with an anti‐phosphotyrosine antibody (1:1000, 9411; Cell Signaling Technology) overnight at 4°C in a blocking solution containing 5% BSA in TBS (Tris‐buffered saline) and 0.1% Tween 20, and subsequently treated with goat anti‐mouse IgG‐HRP (horseradish peroxidase) antibody (1:5000, Santa Cruz Biotechnology) for 2 hrs at room temperature. Blots were developed with enhanced chemiluminescence (GE Healthcare, Hong Kong, China) and exposed on X‐ray film (Fuji Photo Film). The blots were then stripped and reprobed with the anti‐α subunit and anti‐β1 subunit antibodies to determine total channel protein. The film was scanned, imaged by a Bio‐Imaging System (Syngene, Frederick, MD, USA), and the intensity of the bands was analyzed using the GeneTools software (Syngene).

### Solutions and chemicals

Tyrode's solution contained (mM): NaCl 140, KCl 5.4, MgCl_2_ 1, CaCl_2_ 1.8, HEPES 10, and glucose 10 (pH adjusted to 7.3 with NaOH). For whole‐cell recordings, the pipette solution contained (mM) KCl 20, potassium aspartate 110, MgCl_2_ 1, HEPES 10, EGTA 5, GTP 0.1, sodium phosphocreatine 5 and Mg‐ATP 5 (pH adjusted to 7.2 with KOH).

All reagents were obtained from Sigma–Aldrich. Stock solutions were made with dimethyl sulfoxide (DMSO) for genistein (100 mM), AG556 (100 mM) and paxilline (1 mM). The stocks were divided into aliquots and stored at −20°C. The DMSO concentration in the perfusion medium was ≤0.1% (v/v) and had no effect on arteries and membrane currents. Sodium orthovanadate stock solution (200 mM) was made with distilled water, and pH was adjusted to 9.0. Previous studies showed that paxilline (1 μM) remarkably inhibited BK current 27 and genisitein (10 μM) and AG556 (10 μM) significantly inhibited Kv4.3 channels, which were countered by protein tyrosine phosphatase (PTP) inhibitor orthovanadate (1 mM) 18. Therefore, the same concentrations of these compounds were adopted in the present study.

### Statistical analysis

Experimental group data are expressed as means ± standard errors of the mean, and *n* represents the number of cells or the number of assays. Student's two‐tailed *t*‐test was used to evaluate the statistical significance of the differences between two groups. One‐way analysis of variance (anova) with Tukey's *post* test or one‐way anova with Bonferroni's *post* test was used for analyzing multiple groups. Values of *P *<* *0.05 were considered to be statistically significant.

## Results

### Effect of paxilline on BK current in BK‐HEK 293 cells

**Figure 1:**
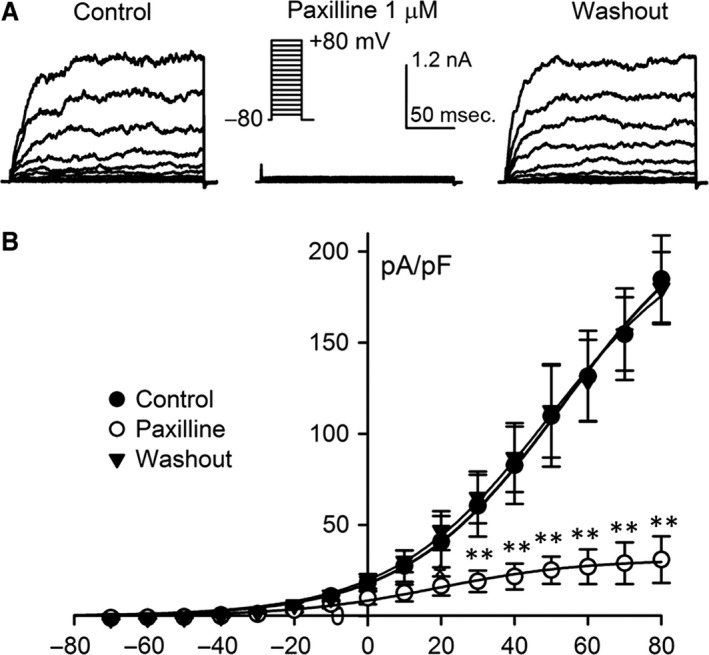
Membrane current in HEK 293 cells stably expressing human BK channel α‐ and β1‐subunits. **(A)** Voltage‐dependent BK current traces recorded in a representative cell with the voltage protocol as shown in the *inset* before and after application of 1 μM paxilline (a selective BK channel blocker), and washout of paxilline. **(B)** *I‐V* relationships of BK current in the absence and presence of 1 μM paxilline, and upon washout (*n* = 5, *P <* *0.05, **P <* *0.01 *versus* control or washout).

Figure 1 illustrates voltage‐dependent current elicited by the voltage steps in BK‐HEK 293 cells stably expressing human BK channel α‐ and β1‐subunits as shown in the inset. The current was sensitive to inhibition by the BK channel blocker paxilline (1 μM, Fig. 1A). The mean values of current‐voltage (*I‐V*) relationships (Fig. 1B) of the current demonstrated a reversible inhibition by paxilline at test potentials from +20 mV to +80 mV, indicating a typical BK current in BK‐HEK 293 cells.

### Effect of genistein on BK current in BK‐HEK 293 cells

**Figure 2:**
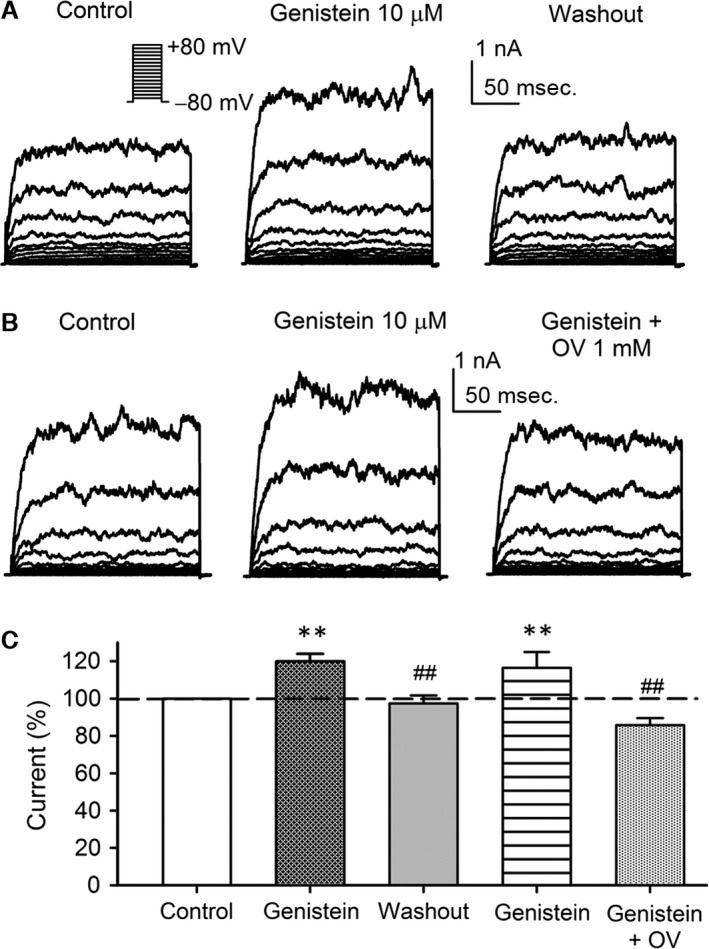
Effect of genistein on BK channel current in BK‐HEK 293 cells. **(A)** Voltage‐dependent BK current recorded in a typical BK‐HEK 293 cell stably expressing both α‐ and β1‐subunit with the voltage protocol as shown in the *inset* in the absence and presence of 10 μM genistein, and upon washout. **(B)** Original BK current traces in a representative BK‐HEK 293 cell during control, in the presence of 10 μM genistein, and genistein plus 1 mM orthovanadate (OV). **(C)** Mean percentage values of BK current measured at +70 mV during control, 10 μM genistein, washout, 10 μM genistein and genistein plus 1 mM OV (*n* = 6, **P <* *0.01 *versus* control; ^##^ *P *<* *0.01 *versus* genistein).

An increase or decrease of BK current by the broad‐spectrum PTK inhibitor genistein was reported in the earlier studies 9, 10. Here we determined how BK current was affected by genistein in BK‐HEK 293 cells. Our results showed that voltage‐dependent BK current was reversibly increased by 10 μM genistein (Fig. 2A). The enhancement effect was fully reversed by co‐application of genistein and the PTP inhibitor orthovanadate (1 mM) (Fig. 2B). Figure 2C illustrates the mean percent values of the BK current measured at +70 mV in control, 10 μM genistein, washout or genistein plus 1 mM orthovanadate. Genistein increased BK current to 116.4% of control, and the effect was reversed by co‐application of orthovanadate. These results suggest that PTK inhibition is involved in BK current increase by genistein.

### Effect of tyrphostin AG556 on BK current in BK‐HEK 293 cells

**Figure 3:**
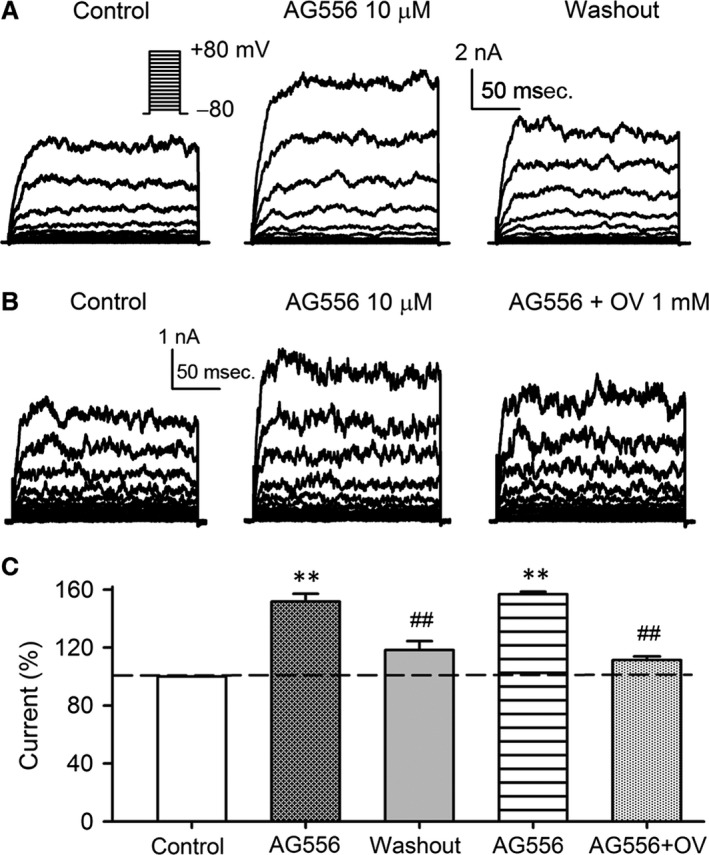
Effect of tyrphostin AG556 (AG556) on BK channel current in BK‐HEK 293 cells. **(A)** Voltage‐dependent BK current recorded in a typical BK‐HEK 293 cell with the voltage protocol as shown in the *inset* in the absence and presence of 10 μM AG556, and upon washout. **(B)** Original BK current traces in a representative BK‐HEK 293 cell during control, in the presence of 10 μM AG556, and AG556 plus 1 mM orthovanadate (OV). **(C)** Mean percentage values of BK current measured at +70 mV during control, 10 μM AG556, washout, 10 μM AG556, and AG556 plus 1 mM OV (*n* = 6, **P <* *0.01 *versus* control; ^##^ *P *<* *0.01 *versus* AG556).

To determine whether EGFR tyrosine kinase is involved in genistein‐induced increase of BK current, AG556, a tyrphostin which selectively inhibited EGFR tyrosine kinase, was tested in BK‐HEK 293 cells. AG556 (10 μM) reversibly increased the voltage‐dependent BK current (Fig. 3A), and the effect was antagonized by co‐application of 1 mM orthovanadate (Fig. 3B). The mean percent value of BK current at +70 mV was increased to 151.8% of control by AG556, and the increase was reversed by washout or application of orthovanadate (Fig. 3C). These results indicate that EGFR tyrosine kinase inhibition may contribute to the increase of BK current by AG556 and genistein.

### Effects of orthovanadate and EGF on BK current in BK‐HEK 293 cells

**Figure 4:**
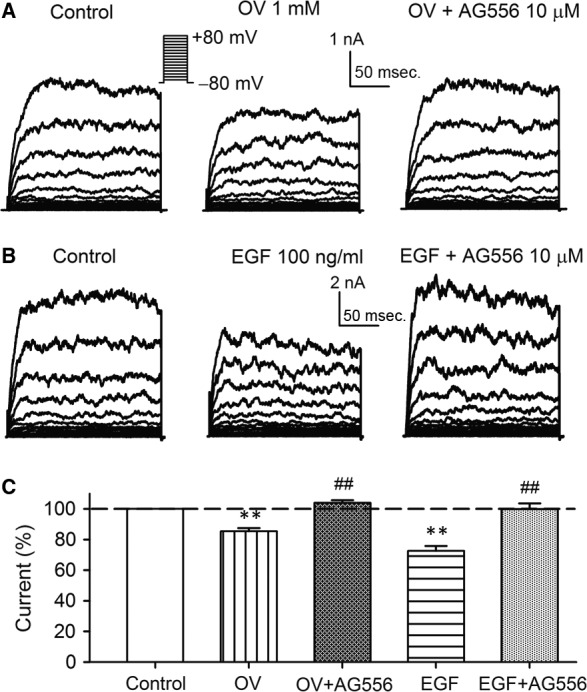
Effects of orthovanadate and EGF on BK channel current in BK‐HEK 293 cells. **(A)** Voltage‐dependent BK current recorded in a representative BK‐HEK 293 cell with the voltage protocol as shown in the *inset* in the absence and presence of 1 mM orthovanadate (OV), and OV plus 10 μM tyrphostin AG556 (AG556). **(B)** Original BK current traces in a representative BK‐HEK 293 cell in the absence and presence of 100 ng/ml EGF, and EGF plus 10 μM AG556. **(C)** Mean percentage values of BK current measured at +70 mV during control, 1 mM OV, OV plus 10 μM AG556, 100 ng/ml EGF and EGF plus 10 μM AG556 (*n* = 5, **P <* *0.01 *versus* control; ^##^ *P *<* *0.01 *versus* OV or EGF alone).

If genistein‐ or AG556‐induced increase of BK current is related to the inhibition of EGFR tyrosine kinase, activation of EGFR kinase by PTP inhibition or EGF application would decrease BK current. Figure 4A shows the voltage‐dependent BK current recorded in a representative BK‐HEK 293 cell in the absence and presence of PTP inhibitor orthovanadate (1 mM), and orthovanadate plus 10 μM AG556. Orthovanadate decreased BK current, and the effect was reversed by AG556. Similarly, the voltage‐dependent BK current was also decreased by EGF (100 ng/ml), and effect was counteracted by co‐application of AG556 (Fig. 4B). Figure 4C illustrates the mean percent values of BK current at +70 mV in control, orthovanadate (1 mM), orthovanadate plus 10 μM AG556, EGF (100 ng/ml) and EGF plus AG556. Orthovanadate decreased the current to 85.4% of control, and the inhibitory effect was fully reversed by co‐application of AG556. BK current was reduced to 72.5% of control by EGF, and the effect was countered by co‐application of AG556. These results support the notion that tyrosine phosphorylation of BK channels by EGFR kinase down‐regulates the channel activity.

### Tyrosine phosphorylation level of BK channel in BK‐HEK 293 cells

**Figure 5:**
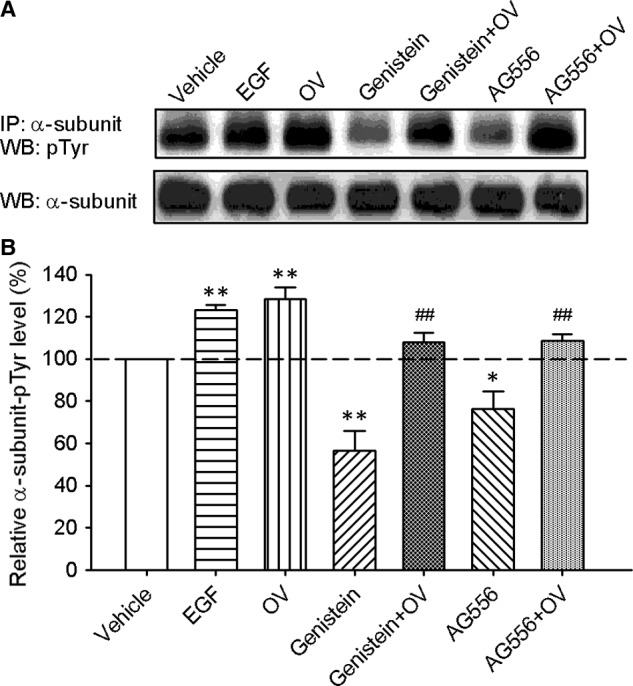
Tyrosine phosphorylation level of α‐subunits in BK‐HEK 293 cells. **(A)** Images of immunoprecipitation and Western blotting in BK‐HEK 293 cells treated with vehicle (control), 100 ng/ml EGF, 1 mM orthovanadate (OV), 10 μM genistein, genistein plus OV, 10 μM AG556 (AG556), and AG556 plus OV. **(B)** Mean percentage values of relative tyrosine‐phosphorylated α‐subunit protein. The amount of protein from the immunoprecipitation (as shown in A) was normalized with those from the Western blotting. Relative phosphorylated protein level is expressed as a percentage of vehicle control (*n* = 6, *P <* *0.05, **P <* *0.01 *versus* control, ^##^ *P *<* *0.01 *versus* genistein or AG556 alone).

To determine the tyrosine phosphorylation level of BK channels, immunoprecipitation and Western blotting analysis were performed in BK‐HEK 293 cells. Figure 5A shows the images of tyrosine phosphorylation level of α‐subunit protein in cells treated with EGF (100 ng/ml), orthovanadate (1 mM), genistein (10 μM), AG556 (10 μM), and genistein or AG556 plus orthovanadate. Figure 5B summarizes the mean values of relative tyrosine phosphorylation levels of α‐subunit protein. The tyrosine phosphorylation of α‐subunit protein was increased by EGF and orthovanadate to 123.1% and 128.3% of control, and reduced to 56.4% and 76.5% of control by genistein and AG556, respectively. The reduction of tyrosine phosphorylation by genistein or AG556 was reversed to 107.5% or 108.7% of control by orthovanadate. These results indicate that the tyrosine phosphorylation level of α‐subunit protein is increased by EGF or tyrosine phosphatase inhibitor and decreased by EGFR kinase inhibition.

**Figure 6:**
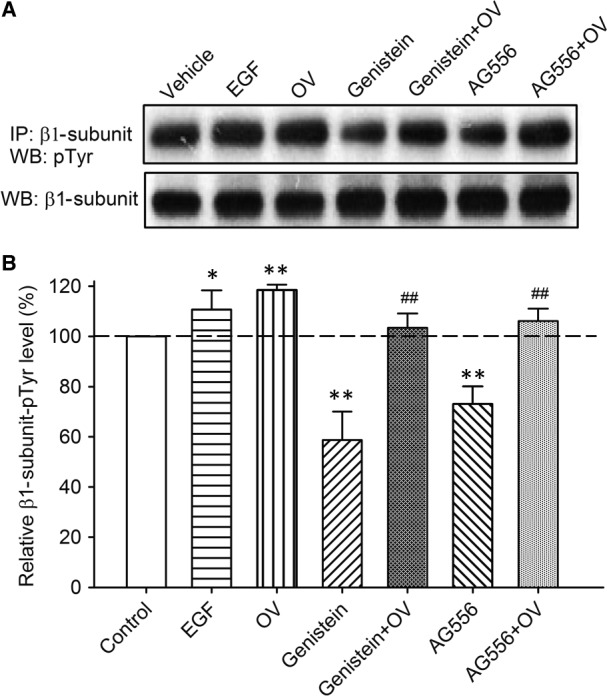
Tyrosine phosphorylation level of β1 subunits in BK‐HEK 293 cells. **(A)** Images of immunoprecipitation and Western blotting in BK‐HEK 293 cells treated with vehicle (control), 100 ng/ml EGF, 1 mM orthovanadate (OV), 10 μM genistein, genistein plus OV, 10 μM tyrphostin AG556 (AG556), and AG556 plus OV. **(B)** Mean percentage values of relative tyrosine‐phosphorylated β1 protein. The amount of protein from the immunoprecipitation (as shown in A) was normalized with those from the Western blotting. Relative phosphorylated protein level is expressed as a percentage of vehicle control (*n* = 5, *P <* *0.05, **P <* *0.01 *versus* control; ^##^ *P *<* *0.01 *versus* genistein or AG556 alone).

Figure 6A displays the images of tyrosine phosphorylation level of β1‐subunits in BK‐HEK 293 cells treated with 100 ng/ml EGF, 1 mM orthovanadate, and 10 μM genistein, 10 μM AG556, or co‐application of 1 mM orthovanadate. The mean percent values of tyrosine phosphorylation level of β1‐subunits were enhanced to 110.7% and 118.5% of control by EGF and orthovanadate, respectively (Fig. 6B). Genistein and AG556 inhibited the phosphorylation level of the β1‐subunit protein respectively by 58.7% and 73.1% of control, and the inhibition was reversed by co‐application of orthovanadate to 103.4% or 106.1% of control (Fig. 6B). These results indicate that the β1‐subunits of BK channels are also phosphorylated by EGFR tyrosine kinase.

### Effect of tyrphostin AG556 on BK current in freshly isolated CASMCs

**Figure 7:**
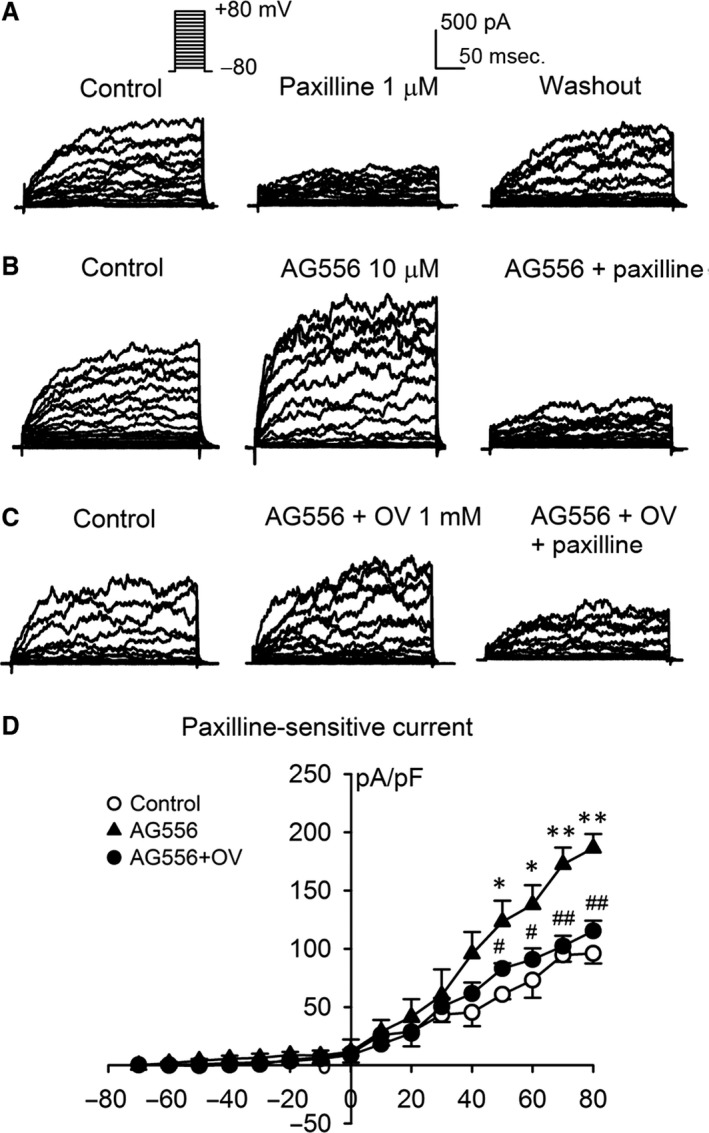
Effect of tyrphostin AG556 (AG556) on BK channel current in freshly isolated cerebral artery smooth muscle cells (CASMCs) from rats. **(A)** Voltage‐dependent outward current recorded with the protocol as shown in the *inset* in a representative CASMC in the absence and presence of 1 μM paxilline, and after washout. **(B)** Membrane current recorded in a typical CASMC in the absence and presence of 10 μM AG556, and AG556 plus 1 μM paxilline. **(C)** Membrane current recorded in a CASMC in the absence and presence of 10 μM AG556 plus 1 mM orthovanadate (OV), and after co‐application of 1 μM paxilline. **(D)** *I‐V* relationships of the mean values of paxilline‐sensitive current obtained by digital subtraction of the current before paxilline application by the current after paxilline application (*n* = 5 for each group, *P <* *0.05, **P <* *0.01 *versus* control; ^#^ *P *<* *0.05, ^##^ *P *<* *0.01 *versus* AG556 alone).

In order to identify the regulation of BK channels by EGFR tyrosine kinase in native cells, we examined the effect of AG556 on BK channel activity in freshly isolated CASMCs. Figure 7A shows the voltage‐dependent outward current elicited by 300‐ms voltage steps from holding potential of −70 mV to test potentials in the range from −80 mV to +80 mV in 10 mV increments (as shown in the inset) in a typical CASMC from rat. The current was reversibly inhibited by BK channel blocker paxilline (1 μM), suggesting that BK channels are predominantly expressed in freshly isolated CASMCs. The membrane current was significantly increased in cells treated with 10 μM AG556, and the increase was antagonized by co‐application of paxilline (Fig. 7B). Interestingly, co‐application of 1 mM orthovanadate and AG556 reduced the effects of AG556 on the current (Fig. 7C). Figure 7D illustrates the *I‐V* relationships of paxilline‐sensitive current obtained by digital subtraction of the current before paxilline by the current after paxilline application in cells without (control) or with AG556, and AG556 plus orthovanadate treatment. The current density at +20 to +80 mV was significantly increased in cells treated with AG556 and partly counteracted by co‐application of AG556 and orthovanadate. These results indicate that the BK current in freshly isolated CASMCs is affected by EGFR tyrosine kinase.

### Effect of tyrphostin AG556 on BK‐mediated vasodilation in rat cerebral basilar arteries

**Figure 8:**
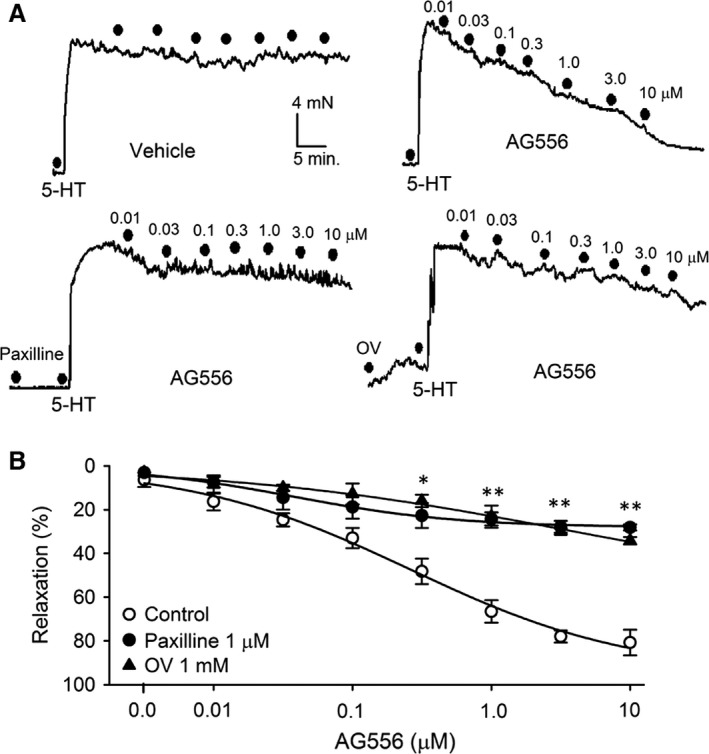
BK‐mediated vasodilation of tyrphostin AG556 (AG556) in rat cerebral basilar arteries. **(A)** Typical records showing concentration‐dependent relaxations induced by AG556 in 1 μM 5‐HT‐precontracted cerebral basilar arteries from rats (dots indicate the concentration of AG556 from 0.01 to 10 μM) in the absence and presence of 1 μM paxilline, or 1 mM orthovanadate (OV). **(B)** Cumulative concentration‐response curves to AG556 in the absence (control) and presence of 1 μM paxilline or 1 mM OV (*n* = 6‐8, *P <* *0.05, **P <* *0.01 *versus* AG556 alone).

To investigate the effect of EGFR kinase inhibition on vascular smooth muscle function, vascular tone was measured in rat endothelium‐denuded cerebral basilar arteries. Figure 8A illustrates the typical traces of vascular contraction induced by 1 μM 5‐HT, which was relaxed by different concentrations of AG556 without or with pretreatment with the BK channel blocker paxilline or the PTP inhibitor orthovanadate, but not by an equivolume of the vehicle DMSO. AG556 exhibited a remarkable vascular relaxation in a concentration‐dependent manner, and the effect was significantly antagonized in arteries pretreated with 1 μM paxilline or 1 mM orthovanadate. It is interesting to note that the pretreatment with orthovanadate induced a slight increase of the vascular tone, likely suggesting an inhibition of BK channels by increasing tyrosine phosphorylation. Figure 8B illustrates the mean percent values of the concentration‐dependent vasodilation by AG556 in the absence (control) or presence of paxilline or orthovanadate. Pretreatment with paxilline to block BK channels or with orthovanadate to inhibit PTPs significantly reduced the vascular relaxation by AG556 in rat arteries. The maximum relaxation efficacy of cerebral basilar arteries by AG556 was decreased by pretreatment with paxilline or orthovanadate. These results indicate that the vasodilation of AG556 is mediated by activating BK channels *via* reducing tyrosine phosphorylation of the channel in rat cerebral basilar arteries, suggesting that the modulation of BK channels by EGFR tyrosine kinase likely play an important role in vascular tone regulation of rat cerebral basilar arteries.

## Discussion

In the present study, we demonstrated that genistein and tyrphostin AG556 increased the activity of BK channels stably expressed in HEK 293 cells, while EGF or the PTP inhibitor orthovanadate decreased the channel activity. Tyrosine phosphorylation levels of both α‐ and β1‐subunits were enhanced by EGF or the PTP inhibitor orthovanadate in BK‐HEK 293 cells, while decreased by genistein and AG556. The changes in BK current and tyrosine phosphorylation levels by genistein and AG556 were counteracted by orthovanadate. In addition, AG556 increased BK current in freshly isolated CASMCs from rats, and dilated the rat cerebral basilar arteries, which was antagonized by the BK channel blocker paxilline or orthovanadate. These results indicate that phosphorylation of BK channels in CASMCs by EGFR tyrosine kinase reduces the channel activity, which may play a key role in regulating physiological vascular tone in cerebral basilar arteries.

It has been reported that EGFR tyrosine kinase participates in regulating different ion channels, including cardiac I_Cl.vol_ channels 29, I_Na_ channels 30, Kv4.3 channels 18, hERG channels 28, I_Ks_ (slowly delayed rectifier K^+^) channels 31, inward‐rectifier K^+^ channels (Kir2.1 and Kir2.3) 19, 32, hEAG1 channels 33 and human SKCa1 (hSKCa1) channels 17. Cardiac native I_Na_ and Kir2.3 channels expressed in HEK 293 cells are increased by EGF (to activate EGFR kinase) or orthovanadate (to inhibit PTPs), but reduced by genistein and AG556 (to inhibit EGFR kinase) 30, 32. However, the basal tyrosine phosphorylation is saturated in most ion channels, thus additional activation is not observed. EGF or orthovanadate does not show any response in hKv4.3 channels 18, hERG channels 28, I_Ks_ 31, hKir2.1 19, hEAG1 channels 33 and hSKCa1 channels 17; nonetheless, the activity of these channels is decreased by PTK inhibitors (genistein and AG556), and the inhibitory effect is significantly counteracted by the PTP inhibitor orthovanadate 17, 18, 19, 28, 31, 33.

The effect of PTKs on BK channels is not fully understood. In a hemorrhagic shock rat model, vascular hyporesponsiveness to norepinephrine is recovered by the application of genistein, and the effect is found to be mediated by increased tyrosine phosphorylation of α‐subunit of BK channels, suggesting an increased BK channel activity by tyrosine phosphorylation in this pathological process 10. In a recent report, genistein (50 μM) induces a PTK‐dependent inhibition of BK current at low level of intracellular free Mg^2+^ (2 μM or 20 μM) but a PTK‐independent increase of the current at high concentration of intracellular free Mg^2+^ (200 μM or 2000 μM) in rat mesenteric arterial smooth muscle cells, suggesting that intracellular Mg^2+^ modulates the effects of genistein on BK channel activity 12. In the present study, genistein and AG556 amplify BK current in BK‐HEK 293 cells at physiological concentration of extracellular Mg^2+^ (1 mM) by inhibiting tyrosine phosphorylation, and the effect of AG556 is further confirmed in freshly isolated cerebral artery smooth muscle cells from rats. One shortage of the present study is that we did not explore whether intracellular Mg^2+^ modulated the effect of AG556 on BK channels in CASMCs.

On the other hand, genistein or tyrphostin A23 (another broad range PTK inhibitor) restores the superoxide‐impaired pig pial artery dilation mediated by BK channels 34. Moreover, genistein reduces the renal arterial contractile response to angiotensin II, norepinephrine, or endothelin‐1 by inhibiting tyrosine kinase 35. The vascular relaxation by PTK inhibition is observed in trabecular meshwork 9 and also rat superior mesenteric arteries 36. The PTP inhibitors orthovanadate and dephostatin induce a reduction of BK current in cells isolated from rat mesenteric arteries 37. A recent study demonstrated that loss of EGFR induces arterial hypotension in a mouse model with targeted deletion of EGFR using smooth muscle‐specific protein 22 promoter 38. These reports suggest that tyrosine phosphorylation of BK channels may decrease the channel activity. The different regulation of BK channels by PTKs may result from the different pathophysiological and/or experimental conditions, such as different vascular beds, various species and Mg^2+^ concentration.

The results from the present study support the notion that phosphorylation of BK channels by EGFR kinase reduces the channel activity, whereas EGFR kinase inhibition by genistein or AG556 increases the channel activity. It is generally recognized that the activation of BK channels results in vasodilation, thus tyrosine phosphorylation of BK channels induces a decrease of channel activity, and this effect likely plays an important role in regulating physiological vascular tone. This is confirmed in the experiment with rat cerebral basilar arteries. AG556 remarkably amplifies BK current in freshly isolated cerebral artery smooth muscle cells from rats, and relaxes the rat cerebral basilar arteries pre‐contracted by 5‐HT *via* inhibiting tyrosine phosphorylation of BK channels, since the effect is significantly antagonized by the PTP inhibitor orthovanadate or the BK blocker paxilline.

In summary, the results from the present study demonstrate the novel evidence that the inhibition of EGFR tyrosine kinase by genistein or AG556 enhances the activity of BK channels, while EGF or the PTP inhibitor decreases the activity of BK channels. Tyrosine phosphorylation of BK channels may play an important role in maintaining physiological vascular tone of cerebral basilar arteries.

## Conflict of interest

The authors confirm that this article content has no conflicts of interest.
